# Immunogenicity and reactogenicity of heterologous ChAdOx1 nCoV-19/mRNA vaccination

**DOI:** 10.1038/s41591-021-01464-w

**Published:** 2021-07-26

**Authors:** Tina Schmidt, Verena Klemis, David Schub, Janine Mihm, Franziska Hielscher, Stefanie Marx, Amina Abu-Omar, Laura Ziegler, Candida Guckelmus, Rebecca Urschel, Sophie Schneitler, Sören L. Becker, Barbara C. Gärtner, Urban Sester, Martina Sester

**Affiliations:** 1 Department of Transplant and Infection Immunology, Saarland University, Homburg, Germany; 2 Department of Internal Medicine IV, Saarland University, Homburg, Germany; 3 Institute of Medical Microbiology and Hygiene, Saarland University, Homburg, Germany

**Keywords:** Vaccines, Viral infection

## Abstract

Heterologous priming with the ChAdOx1 nCoV-19 vector vaccine followed by boosting with a messenger RNA vaccine (BNT162b2 or mRNA-1273) is currently recommended in Germany, although data on immunogenicity and reactogenicity are not available. In this observational study we show that, in healthy adult individuals (*n* = 96), the heterologous vaccine regimen induced spike-specific IgG, neutralizing antibodies and spike-specific CD4 T cells, the levels of which which were significantly higher than after homologous vector vaccine boost (*n* = 55) and higher or comparable in magnitude to homologous mRNA vaccine regimens (*n* = 62). Moreover, spike-specific CD8 T cell levels after heterologous vaccination were significantly higher than after both homologous regimens. Spike-specific T cells were predominantly polyfunctional with largely overlapping cytokine-producing phenotypes in all three regimens. Recipients of both the homologous vector regimen and the heterologous vector/mRNA combination reported greater reactogenicity following the priming vector vaccination, whereas heterologous boosting was well tolerated and comparable to homologous mRNA boosting. Taken together, heterologous vector/mRNA boosting induces strong humoral and cellular immune responses with acceptable reactogenicity profiles.

## Abstract (web summary)

In healthy adults, booster vaccination with an mRNA vaccine, irrespective of the vaccine used for the first dose, was well tolerated and elicited higher levels of spike-specific antibodies and spike-specific T cells than booster vaccination with ChAdOx1 nCov-19.

## Main

Among the currently authorized COVID-19 vaccines, the ChAdOx1 nCoV-19 adenovirus-based vector vaccine (ChAdOx1) and the two mRNA vaccines (BNT162b2 and mRNA-1273) have been the most widely used. Both vaccine types are immunogenic and have shown remarkable efficacy in preventing COVID-19 disease^1–3^. In March 2021, administration of the ChAdOx1 vaccine was temporarily suspended in Germany due to the occurrence of life-threatening cerebral venous thrombosis and thrombocytopenia, primarily in younger women^4,5^. This resulted in revised recommendations for secondary vaccination of all individuals who had received the first dose of the vaccine^6^. Individuals above the age of 60 years are recommended to complete vaccination with the vector vaccine, whereas heterologous boosting with an mRNA vaccine is recommended in those <60 years, with the option to voluntarily remain on a homologous vector regimen^6^. Comparative analyses of immunogenicity between the authorized vaccine regimens are scarce, and knowledge on immunity and reactogenicity after heterologous vaccination is currently limited. We have found that priming with the ChAdOx1 vaccine showed a stronger induction of spike-specific T cell responses as compared to mRNA priming, while antibody responses were more pronounced after mRNA priming^7^. We hypothesized that differences among the vaccine types after priming may influence cellular and humoral immunity following secondary vaccination. We therefore prospectively enrolled three groups of individuals to study the immunogenicity and reactogenicity of a heterologous vector/mRNA prime–booster regimen in comparison to the standard homologous regimens. A detailed analysis of spike-specific IgG levels and neutralizing antibody activity was performed. In addition, spike-specific CD4 and CD8 T cells were characterized using flow cytometry. Adverse events within the first week after the priming and booster doses were self-reported based on a standardized questionnaire.

**Extended Data Fig. 1:**
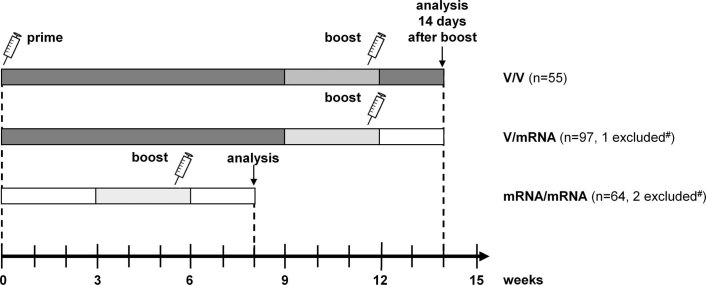
Study design. Time between first and second vaccination and the time between second vaccination and analysis is shown for the three vaccination regimens (homologous ChAdOx1 nCoV-19 vector vaccination (V/V, n = 55), heterologous ChAdOx1 nCoV-19 vector/mRNA vaccination (V/mRNA, n = 97) or homologous mRNA vaccination (mRNA/mRNA, n = 64). ^#^3 individuals were excluded from further analysis due to IgG positivity in a SARS-CoV-2 nucleocapsid ELISA.

**Extended Data Fig. 2:**
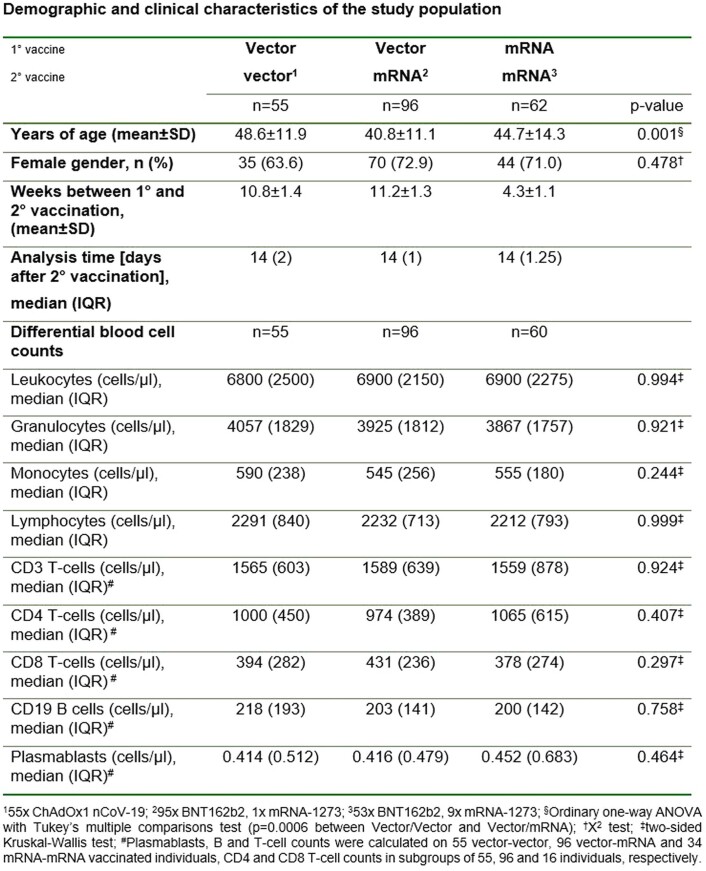
Demographic and clinical characteristics of the study population. Demographic characteristics and vaccine-related data are shown for the three vaccination regimens (homologous ChAdOx1 nCoV-19 vector vaccination (V/V, n = 55), heterologous ChAdOx1 nCoV-19 vector/mRNA vaccination (V/mRNA, n = 96) or homologous mRNA vaccination (mRNA/mRNA, n = 62)). In addition, information on differential blood counts and on lymphocyte subpopulations is provided.

A total of 216 immunocompetent individuals, primarily comprising employees, were prospectively enrolled at Saarland University Medical Center before secondary vaccination with the authorized vaccines ChAdOx1 nCoV-19, BNT162b2 or mRNA-1273 (Methods). Ninety-seven study participants received heterologous vaccination with the ChAdOx1 vector and mRNA booster (vector/mRNA), whereas 55 and 64 received homologous regimens with vector or mRNA vaccine, respectively (vector/vector and mRNA/mRNA; Extended Data Fig. 1). As per guidelines, the time between primary and secondary vaccination was shorter for mRNA-primed (4.3 ± 1.1 weeks) than for vector-primed individuals, with no difference between vector-based heterologous (11.2 ± 1.3 weeks) and homologous regimens (10.8 ± 1.4 weeks). Blood samples were drawn at a median of 14 (interquartile range (IQR) = 2) days after vaccination. Although all individuals had no known history of SARS-CoV-2 infection, three tested positive for SARS-CoV-2 nucleocapsid (N)-specific IgG and were excluded from further analyses. The groups had similar gender distribution. However, individuals on the homologous vector regimen were slightly older than the two other groups, who were of similar age (Extended Data Fig. 2). Leukocyte counts, including granulocytes, monocytes and lymphocytes, as well as major lymphocyte subpopulations such as CD4 and CD8 T cells, and B cells, did not differ between the groups. This also held true for plasmablasts, which were identified as CD38-positive cells among IgD^–^CD27^+^ CD19-positive switched-memory B cells (Extended Data Fig. 2).

**Fig. 1:**
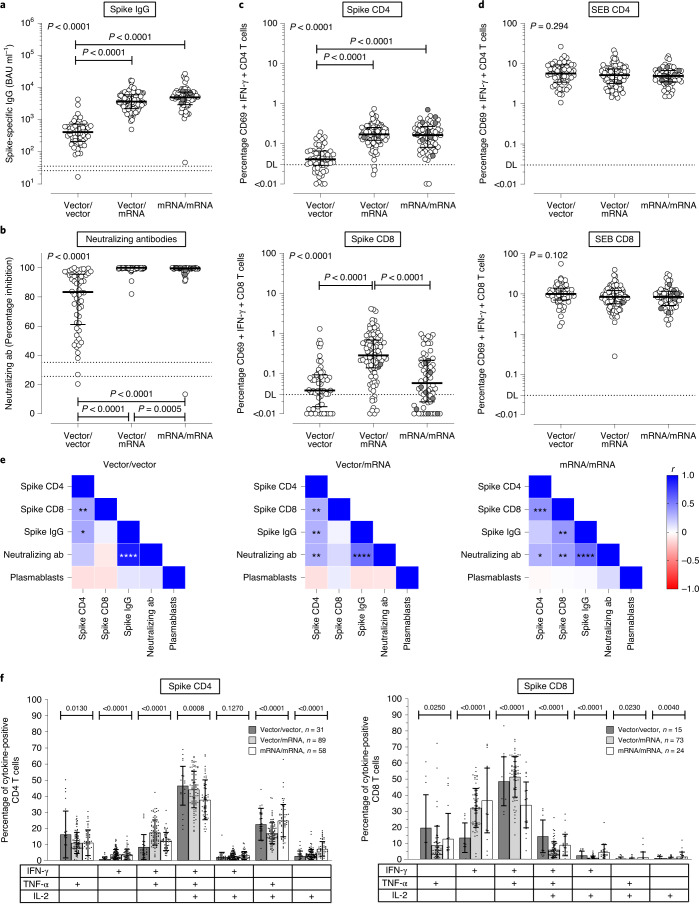
Immune responses against the SARS-CoV-2 spike protein after vaccination with homologous and heterologous prime–booster regimens. Immune responses were compared between individuals who received either homologous ChAdOx1 nCoV-19 vector/vector vaccination (*n* = 55), heterologous ChAdOx1 nCoV-19 vector/mRNA vaccination (*n* = 96) or homologous mRNA/mRNA vaccination (*n* = 62). **a**,**b**, Spike-specific IgG levels (**a**) and neutralizing antibodies (ab) (**b**) were quantified by ELISA and a surrogate neutralization assay. **c**,**d**, Percentages of SARS-CoV-2 spike-specific (**c**) and SEB-reactive (**d**) CD4 and CD8 T cells were determined after antigen-specific stimulation of whole-blood samples, followed by intracellular cytokine analysis using flow cytometry. Reactive cells were identified by coexpression of CD69 and IFN-γ among CD4 or CD8 T cells and subtraction of background reactivity of respective negative controls. **e**, Correlations between spike-specific T cell levels, antibody responses and numbers of plasmablasts. **f**, Cytokine expression profiles of spike-specific CD4 and CD8 T cells in all individuals showing single or combined expression of IFN-γ, IL-2 and TNF-α (gating strategy shown in Extended Data Fig. 5). **a**–**d**, Bars represent medians with IQR. Individuals who received the mRNA-1273 vaccine are indicated in gray (1x vector/mRNA, 9x mRNA/mRNA). Differences between groups were calculated using a two-sided Kruskal–Wallis test with Dunn´s multiple comparisons post test. **e**, Correlation coefficients (*r*) were analyzed according to two-tailed Spearman (Supplementary Table 1). **f**, Bars represent means and standard deviation; ordinary one-way ANOVA tests were performed. **a**,**b**, Dotted lines indicate detection limits (DL) for antibodies, indicating negative, intermediate and positive levels or levels of inhibition, respectively, per the manufacturer’s instructions. **c**,**d**, Dotted lines indicate detection limits for SARS-CoV-2-specific CD4 T cells. **f**, Analysis was restricted to samples with ≥30 cytokine-positive T cells (*n* = 31 (CD4) and *n* = 15 (CD8) for vector/vector, *n* = 89 and *n* = 73 for vector/mRNA and *n* = 58 and *n* = 24 for mRNA/mRNA).

Spike-specific IgGs were induced in 212/213 individuals after vaccination. IgG levels after heterologous vaccination and homologous mRNA vaccination were similar (3,630 (IQR = 3721) and 4,932 (IQR = 4,239) BAU ml^–1^, respectively), whereas levels after homologous vector vaccination were significantly lower (404 (IQR = 510) BAU ml^–1^, *P* < 0.0001, two-sided Kruskal–Wallis test with Dunn´s multiple comparisons post test; Fig. 1a). This difference was also observed for neutralizing antibodies, which were quantified by a surrogate neutralization test. While the majority of individuals in the vector/mRNA and mRNA/mRNA groups had 100% inhibitory activity, this was significantly lower in the vector/vector group (Fig. 1b).

**Extended Data Fig. 3:**
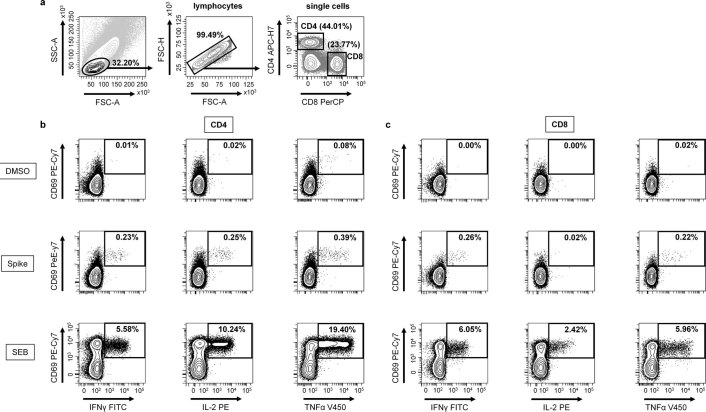
Representative analysis of spike- and SEB-reactive CD4 and CD8 T cells. Lymphocytes were identified among total events by backgating of CD4 and/or CD8 positive cells combined with signals for size (FSC) and granularity (SSC). Hight and area signals of FSC were used to exclude doublets. CD4 T cells were identified among single cells by CD4 positive and CD8 negative signals. Likewise, CD8 T cells were defined as T cells being CD8 positive and CD4 negative. In (**b**) and (**c**) representative contour plots of CD4 and CD8 T cells of a 37-year-old female are shown after antigen-specific stimulation with SARS-CoV-2 spike peptides or respective control stimuli for negative (DMSO) or positive control (SEB) stimulation. Numbers indicate percentages of CD4 or CD8 T cells co-expressing the activation marker CD69 and the cytokines IFN-γ, IL-2 and/or TNF-α. FSC, forward scatter; IFN, interferon; IL, interleukin; SEB, Staphylococcus aureus enterotoxin B; SSC, side scatter; TNF, tumor necrosis factor.

**Extended Data Fig. 4:**
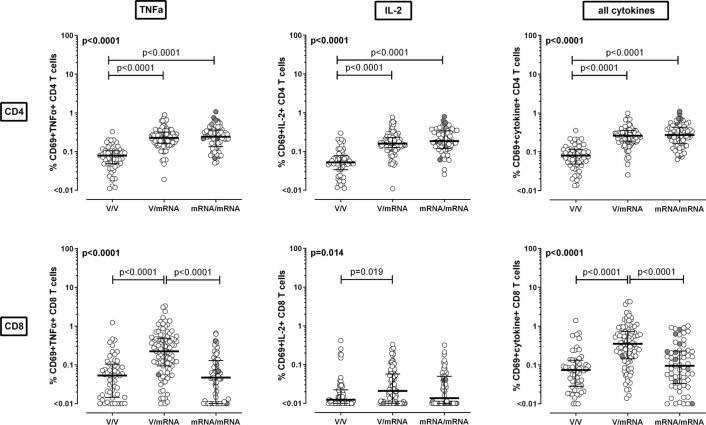
SARS-CoV-2 spike-specific T cells producing TNF-α and IL-2 after vaccination with homologous and heterologous prime-booster regimens. Percentages of SARS-CoV-2 spike-specific CD4 and CD8 T cells were determined after antigen-specific stimulation of whole blood samples followed by intracellular cytokine analysis using flow-cytometry. Reactive cells were identified by co-expression of the activation marker CD69 and the cytokine tumor necrosis factor (TNF) α (left panel), interleukin 2 (IL-2, middle panel), or any of the cytokines analyzed (IFN-γ/TNF-α/IL-2, right panel), respectively, among CD4 or CD8 T cells and subtraction of background reactivity of respective negative control stimulations. T cell responses were compared between individuals who either received SARS-CoV-2 vector/vector (V/V, n = 55), vector/mRNA (V/mRNA, n = 96) or mRNA/mRNA vaccines (mRNA/mRNA, n = 62) using two-sided Kruskal-Wallis test with Dunn’s multiple comparisons post test. Bars represent medians with interquartile ranges, individuals who received the mRNA-1273 vaccine are indicated by grey symbols.

In the analysis of vaccine-induced T cell responses, overlapping peptide pools derived from the spike protein were used to stimulate whole-blood samples (Methods). Spike-specific CD4 and CD8 T cells were identified using flow cytometry by induction of CD69 and the cytokines interferon (IFN)-γ, tumor necrosis factor (TNF)-α and interleukin (IL)-2. The gating strategy and representative contour plots of cytokine-positive CD4 and CD8 T cells from a 37-year-old woman following heterologous vaccination are shown in Extended Data Fig. 3. Both the heterologous vector/mRNA and homologous mRNA/mRNA regimen led to a marked induction of spike-specific, IFN-γ-producing CD4 T cells with median percentages of 0.17 (IQR = 0.13%) and 0.16 (IQR = 0.19%), respectively, whereas CD4 T cell levels following homologous vector/vector vaccination were significantly lower (median 0.04% (IQR = 0.04%), each *P* = 0.0001; Fig. 1c). Interestingly, heterologous mRNA boosting in vector-primed individuals induced the highest percentages of spike-specific IFN-γ-producing CD8 T cells (0.28% (IQR = 0.54%)), which were not only more pronounced than after homologous vector boosting (vector/vector, 0.04% (IQR = 0.08%)) but also higher than for the mRNA/mRNA regimen (0.06% (IQR = 0.19)%, *P* < 0.0001; Fig. 1c). *Staphylococcus aureus* enterotoxin B (SEB)-reactive CD4 or CD8 T cell levels did not differ between groups (Fig. 1d). As with IFN-γ-producing CD4 and CD8 T cells, similar between-group differences were found for spike-specific CD4 T cells producing TNF-α or IL-2, and for spike-specific CD8 T cells producing TNF-α (Extended Data Fig. 4). Because CD8 T cells generally produce less IL-2, differences were less pronounced for IL-2-producing CD8 T cells. Finally, between-group differences were similar if CD4 or CD8 T cells producing any of the three cytokines were considered after Boolean gating (Extended Data Fig. 4).

An overview summarizing correlations between plasmablasts and spike-specific IgG and their neutralizing activity, and spike-specific CD4 and CD8 T cells, is shown in Fig. 1e and Supplementary Table 1. As expected, IgG levels showed a significant correlation with neutralizing activity in all three groups. A strong correlation was also found between spike-specific CD4 and CD8 T cell levels. In line with the role of CD4 T cells in supporting antibody production, CD4 T cells correlated with IgG levels in both the vector/vector and vector/mRNA groups. In the mRNA/mRNA group, antibody levels and neutralizing activity were found to correlate with CD8 T cell levels; whether this reflects a causal relationship or similar induction kinetics is currently unknown.

**Extended Data Fig. 5:**
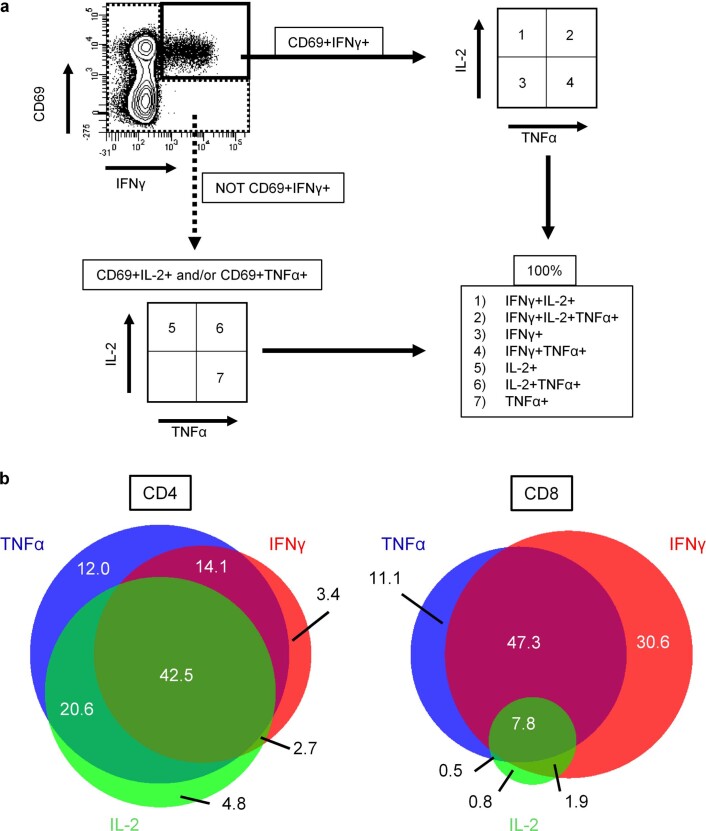
Gating strategy and analysis of spike-specific cytokine-expression profiles. (**a**) To characterize spike- and SEB-reactive CD4 T cells regarding their single or combined expression of the cytokines IFN-γ, IL-2, and TNF-α, CD4 T cells positive for combined expression of CD69 and IFN-γ were divided into four subpopulations according to additional expression of IL-2 and TNF-α. Using NOT Boolean Gating, all CD4 T cells which were not CD69 + IFN-γ + were analyzed for CD69 + IL-2+ and CD69 + TNF-α + CD4 T cells. Using OR Boolean Gating, CD69 + IL-2+ and/or CD69 + TNF-α + CD4 T cells were divided into IL-2 single, TNF-α single or IL-2+TNF-α + cells. After subtraction of background reactivity from negative control stimulations, the sum of these 7 subpopulations was set to 100%. A similar strategy was applied for CD8 T cells. (**b**) Cytokine-expression profiles of spike-specific CD4 and CD8 T cells in all individuals showing single or combined expression of the cytokines IFN-γ, interleukin (IL) 2 and tumor necrosis factor (TNF) α. To allow for robust statistics, analysis was restricted to samples with at least 30 cytokine-positive T cells (n = 178 for CD4 and n = 112 for CD8 T cells). Numbers refer to the percentage of cells expressing the respective cytokine. IFN, interferon; IL, interleukin; TNF, tumor necrosis factor.

**Extended Data Fig. 6:**
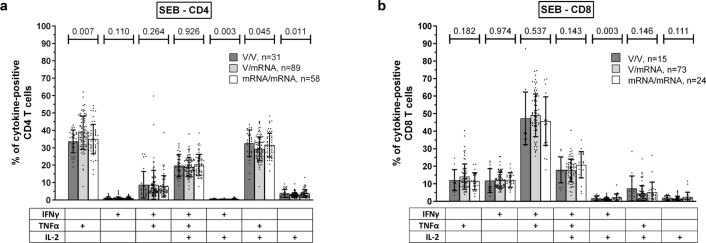
Cytokine-expression profiles of SEB-reactive CD4 and CD8 T cells after vaccination with homologous and heterologous prime-booster regimens. Cytokine expression of CD4 (**a**) and CD8 T cells (**b**) after stimulation with Staphylococcus aureus enterotoxin B (SEB), was compared between individuals who either received SARS-CoV-2 vector/vector (V/V), vector/mRNA (V/mRNA), or mRNA/mRNA vaccine combinations. Cytokine-expressing T-cells were divided into 7 subpopulations according to their expression of IFN-γ, IL-2, and TNF-α (single, double or triple cytokine-expressing cells, for gating strategy, see Extended Data Fig. 5). Only samples of the study participants included in Fig. 1f are displayed (at least 30 cytokine-expressing CD4 or CD8 T-cells, respectively, after spike-specific stimulation, n = 31 and n = 15 for V/V, n = 89 and n = 73 for V/mRNA and n = 58 and n = 24 for mRNA/mRNA vaccine regimes). Differences among subpopulations between the groups were determined using ordinary one-way ANOVA test. Bars represent means and standard deviations of subpopulations among all SEB-reactive CD4 and CD8 T cells, respectively. IFN, interferon, IL, interleukin, TNF, tumor necrosis factor.

In addition to quantitative analysis of spike-specific CD4 and CD8 T cells, we also characterized the cytokine profiles of IFN-γ, IL-2 and TNF-α at the single-cell level. Based on the gating strategy shown in Extended Data Fig. 5, seven subpopulations—including multifunctional T cells producing all three cytokines—could be distinguished. When analyzed across all three participant groups, the majority of spike-specific CD4 T cells (42.5%) were multifunctional. In contrast, the dominant population among CD8 T cells consisted of dual-positive cells producing IFN-γ and TNF-α (47.3%), followed by 30.6% IFN-γ single-positive cells (Extended Data Fig. 5). The distribution of cytokine-producing cells showed significant differences between the three participant groups, which held true for both spike-specific CD4 and CD8 T cells (Fig. 1f). Given the particular ability of the vector vaccine to induce spike-specific T cells during priming^7^, it is notable that the two vector-primed groups had the highest percentage of polyfunctional CD4 T cells irrespective of the boosting vaccine. This also held true for the dominant fraction of CD8 T cells coproducing IFN-γ and TNF-α. In contrast, SEB-reactive cytokine profiles among CD4 and CD8 T cells were similar in all vaccine groups (Extended Data Fig. 6).

**Fig. 2:**
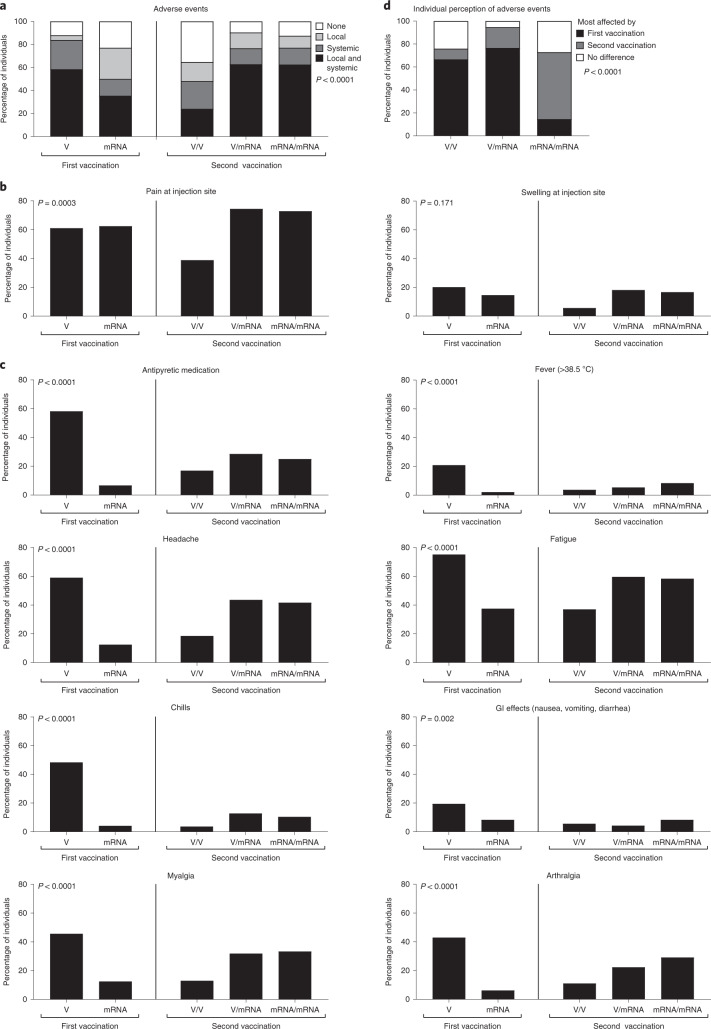
Reactogenicity after primary and secondary vaccination with homologous and heterologous prime–booster regimens. **a**–**c**, Reactogenicity within the first week after priming and after the booster dose was self-reported based on a standardized questionnaire, and was analyzed after the first vaccination with either vector (V, *n* = 150) or mRNA vaccine (mRNA, *n* = 48), and after the second vaccination with homologous (V/V, *n* = 54; mRNA/mRNA, *n* = 48) and heterologous (V/mRNA, *n* = 95) vaccine regimens with respect to local/systemic reactions in general (**a**), stratified for local (**b**) and various systemic adverse events (**c**). **d**, Individual perception of which of the two vaccinations had the greater affect. Comparisons between groups were performed using the *X*² test. GI, gastrointestinal.

Finally, local and systemic adverse events within the first week after primary and secondary vaccination were recorded using a questionnaire (Fig. 2a). Local reactions, such as pain at the injection site and swelling, were similar after priming with the vector and mRNA vaccines (Fig. 2b). However, participants reported significantly more systemic adverse events, including fever, chills, gastrointestinal events, headache, fatigue, myalgia or arthralgia, after the vector vaccine; participants also reported more frequent use of antipyretic drugs (Fig. 2c). When comparing reactogenicity after secondary vaccination, both local and systemic events were markedly less frequent in vector-primed individuals after the second vector booster. Boosting with an mRNA vaccine was less well tolerated in both vector- and mRNA-primed individuals, and the spectrum of local and systemic adverse events was very similar for both groups. Individual perception after the first or the second dose appears to be determined by the severity of the priming vector vaccine, as recipients of both the homologous vector and the heterologous vector/mRNA regimen were most affected after the first vaccination (Fig. 2d).

Mixing different vaccine types in heterologous regimens has already been deployed in previous vaccine studies. Examples include experimental vaccines towards human immunodeficiency virus^8^ and malaria^9^, and the authorized vector vaccine against Ebola virus disease^10^. Although no data were available on the immunogenicity and efficacy of heterologous strategies among authorized COVID-19 vaccines, this raised confidence in recommending a heterologous mRNA booster vaccination in ChAdOx1 vector-primed individuals after recognition of severe adverse events of cerebral venous thrombosis^4,5^. We show that the heterologous regimen led to a strong induction of both antibodies and T cells. IgG levels were similar in magnitude to those following homologous mRNA vaccination, and approximately tenfold higher than those after homologous vector vaccination. Similar differences were found for vaccine-induced CD4 T cells, while neutralizing antibody activity and spike-specific CD8 T cell numbers were even more pronounced after heterologous vaccination. Similar results were recently reported in mice^11^. Despite the strong ability of the ChAdOx1 vaccine to induce T cells after priming^7^, the strikingly lower immunogenicity following the homologous ChAdOx1 booster dose affected both antibodies and T cells. This may result from neutralizing immunity towards the vector backbone induced after the first vaccine dose^12^, which could have rendered secondary vaccination less efficient. Our results show that both vector-primed antibodies and T cells are particularly well induced when combined with mRNA as the secondary vaccine.

**Extended Data Fig. 7:**
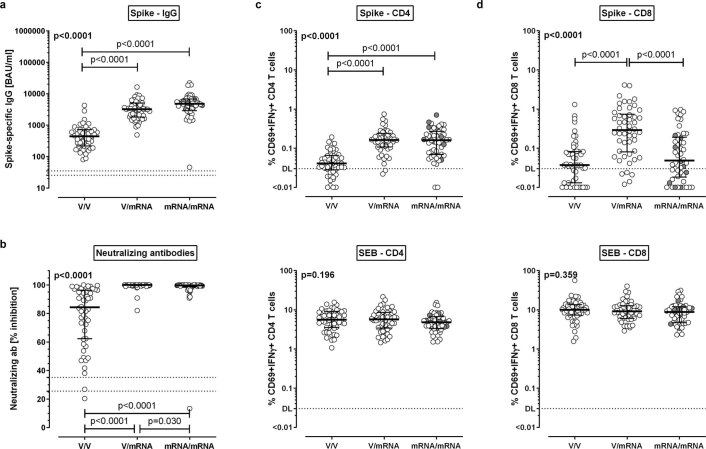
Immune responses against the SARS-CoV-2 spike protein after vaccination with homologous and heterologous prime-booster regimens in age-matched subgroups. Immune responses were compared between age-matched subgroups of 50 individuals each who either received homologous ChAdOx1 nCoV-19 vector vaccination (V/V, mean age 47.0 ± 11.2 years, 33 females), heterologous ChAdOx1 nCoV-19 vector/mRNA vaccination (V/mRNA, 47.1 ± 9.2 years, 38 females) or homologous mRNA vaccination (mRNA/mRNA, 46.4 ± 11.5 years, 35 females, p = 0.940). Spike-specific IgG-levels (**a**) and neutralizing antibodies (**b**) were quantified by ELISA and a surrogate neutralization assay and compared between groups. Percentages of SARS-CoV-2 spike-specific (**c**) and SEB-reactive (**d**) CD4 and CD8 T cells were determined after antigen-specific stimulation of whole blood samples followed by intracellular cytokine analysis using flow-cytometry. Reactive cells were identified by co-expression of CD69 and the cytokine interferon (IFN) γ among CD4 or CD8 T cells and subtraction of background reactivity of respective negative control stimulations. Bars represent medians with interquartile ranges. Individuals who received the mRNA-1273 vaccine are indicated by grey symbols (9 in the homologous mRNA/mRNA group). Differences between the groups were calculated using two-sided Kruskal-Wallis test with Dunn´s multiple comparisons post test. Dotted lines indicate detection limits for antibodies in (a) and (b), indicating negative, intermediate and positive levels or levels of inhibition, respectively as per manufacturer’s instructions, and detection limits for SARS-CoV-2-specific CD4 T cells in (c) and (d). SEB, Staphylococcus aureus enterotoxin B.

Although the heterologous group reported more pronounced systemic adverse events after vector priming, boosting with the mRNA vaccine was less severe and well tolerated and the spectrum of both local and systemic adverse events was comparable to the homologous mRNA regimens. A recent study also showed strong induction of humoral immunity after a second dose of BNT162b2 in individuals primed with the vector vaccine. Similar to our study, the booster dose was given 8–12 weeks after priming^13^. Although there was no direct comparison with homologous regimens, the reactogenicity profile was also rather moderate^13^. This contrasts with reactogenicity data from the Com-COV trial, where adverse events in the heterologous regimens were more severe than in homologous groups^14^. Whether this was due to the shorter interval between priming and boosting (4 versus 9–12 weeks) and whether this affects immunogenicity await further study. Our observational study based on a convenience cohort is limited by the fact that direct comparison of immunity in the same individuals after the first vaccine dose was not possible, because data after primary vaccination were available for only a subset of the mRNA/mRNA group and the majority of vector-primed individuals were enrolled after primary vaccination. However, in a separate group of vector-primed individuals, antibody levels were higher following mRNA priming whereas T cell levels were higher after vector priming^7^. Another limitation of our study is that most mRNA vaccine recipients received BNT162b2, although results appear similar for mRNA-1273. In addition, the homologous ChAdOx1 nCoV-19 vaccine group was slightly older due to age-related differences in general recommendations. However, because a subgroup analysis of age-matched individuals gave the same results (Extended Data Fig. 7), we speculate that the difference in age is unlikely to account for the reduced immune responses in this group. Although we show strong neutralizing activity in a surrogate assay, neutralizing activity towards wild-type virus or variants of concern was not specifically assessed. Finally, no vaccine efficacy data are available to inform on protection from infection or disease. While this awaits further study, immune-based correlates of protection will be important in estimating the efficacy of vaccines and vaccine combinations^15^. Neutralizing antibodies have been discussed as promising candidates^15,16^ that mirror the efficacy of vector and mRNA regimens^1–3^. Because immunogenicity after heterologous vaccination is comparable—or in part superior—to homologous mRNA regimens, it will be interesting to see whether this translates into similar efficacy.

Although vaccine development focuses on antibodies due to their ability to confer sterilizing immunity, T cells are important in mediating protection from severe disease^17^ and may be less affected by virus variants^18^. The T cell data from this and similar studies could influence the development of future vaccine strategies, including how to improve vaccine-induced T cell immunity and protection from severe disease among vulnerable groups of immunocompromised patients.

## Methods

### Study design and subjects

A convenience cohort of immunocompetent individuals with no known history of SARS-CoV-2 infection was invited to participate in this observational study, and were enrolled before secondary vaccination and after primary vaccination with either ChAdOx1 nCoV-19 or one of the mRNA vaccines (BNT162b2 or mRNA-1273). The majority of study participants were PCR tested on a regular basis due to their occupational activity in a hospital setting. This study was not a randomized clinical trial, but was based on the revised recommendations that were issued in Germany on 1 April 2021 for secondary vaccination of all individuals who had received the first dose of the ChAdOx1 nCoV-19 vector vaccine^6^. The study did not influence the decision on vaccine regimens: decisions were exclusively assigned based on current recommendations^6^. Blood sampling for immunological analyses was performed after having received a homologous vaccine regimen comprising either ChAdOx1 nCoV-19 or one of the mRNA vaccines (BNT162b2 or mRNA-1273), or a heterologous vaccine regimen comprising a ChAdOx1 nCoV-19 priming dose followed by secondary vaccination with an mRNA vaccine. The time interval between the first and second dose was determined as per national guidelines^6^ and varied from 3 to 6 weeks for the homologous mRNA regimens to 9–12 weeks for the homologous ChAdOx1 nCoV-19 and the heterologous ChAdOx1 nCoV-19/mRNA regimena^19^. Lymphocyte subpopulations, as well as vaccine-induced SARS-CoV-2-specific humoral and cellular immunity, were determined from heparinized whole blood 14 days after the second vaccine dose, with an interval of 13–18 days tolerated. Local and systemic adverse events within 7 days after the first and second vaccination were self-reported using a standardized questionnaire. Reactogenicity after the second dose was collected prospectively in all cases using a standardized questionnaire. Reactogenicity data after the first dose were collected retrospectively in the majority of cases, but all participants felt confident in recalling adverse events at the time of enrollment into the study.

Vaccinations were performed between 10 January and 8 April 2021. All individuals in the vector-primed group had their first vaccination before 1 April, because primary vector vaccination was suspended at Saarland University Medical Center thereafter. Immunity after secondary vaccination in 28 individuals in the mRNA/mRNA group was tested before 1 April. Testing in these individuals was offered as a service to those working in intensive care units on a voluntary basis as part of routine diagnostics. All provided written informed consent to have their immunogenicity and reactogenicity data included as control group in this study. Moreover, 32 individuals (20 mRNA and 12 vector primed) represent a subgroup of immunocompetent controls enrolled in a separate observational study (SaarTxVac study). Their results following the induction of humoral and cellular immunity after the first vaccination are part of a separate manuscript^7^ that addresses the induction of humoral and cellular immunity after vector and mRNA priming in immunocompetent individuals and transplant recipients. The results of their immune response after secondary vaccination (18 cases tested before and 14 after 1 April) are included in the present study. The study was approved by the ethics committee of the Ärztekammer des Saarlandes (no. 76/20), and all individuals gave written informed consent.

### Quantification of lymphocyte populations and plasmablasts

T cells, B cells and plasmablasts were quantified from 100 µl of heparinized whole blood as described previously^20^ using monoclonal antibodies towards CD3 (clone SK7, final dilution 1:25), CD19 (clone HIB19, 1:40), CD27 (clone L128, 1:200), CD38 (clone HB7, 1:20) and IgD (clone IA6-2, 1:33.3). T and B cells were identified among total lymphocytes by expression of CD3 and CD19, respectively. Plasmablasts were characterized by expression of CD38 among IgD^–^CD27^+^ CD19-positive switched-memory B cells. CD4 and CD8 T cells were quantified after additional staining of CD4 (clone SK3, 1:100) and CD8 (clone RPA-T8). Antibodies used are listed in Supplementary Table 2. Analysis was performed on a BD FACSLyric flow-cytometer and BD FACSuite software v.1.4.0.7047, followed by data analysis using FlowJo software 10.6.2. The gating strategy is shown in Supplementary Fig. 1. Absolute lymphocyte numbers were calculated based on differential blood counts.

### Quantification of vaccine-induced SARS-CoV-2-specific T cells

SARS-CoV-2-specific T cells were determined from heparinized whole blood after 6-h stimulation with overlapping peptides spanning the SARS-CoV-2 spike protein (N-terminal receptor binding domain and C-terminal portion including the transmembrane domain, each peptide 2 µg ml^–1^; JPT) as described previously^20^. Stimulations with 0.64% DMSO and 2.5 μg ml^–1^ of SEB (Sigma) served as negative and positive controls, respectively. All stimulations were carried out in the presence of costimulatory antibodies against CD28 and CD49d (clones L293 and 9F10, 1 μg ml^–1^ each). Immunostaining was performed using anti-CD4 (clone SK3, 1:33.3), anti-CD8 (clone SK1, 1:12.5), anti-CD69 (clone L78, 1:33.3), anti-IFN-γ (clone 4 S.B3, 1:100), anti-IL-2 (clone MQ1-17H12, 1:12.5) and anti-TNF-α (clone MAb11, 1:20), and analyzed using flow cytometry (BD FACS Canto II, including BD FACSdiva software 6.1.3). Antibodies are listed in Supplementary Table 2. SARS-CoV-2-reactive CD4 or CD8 T cells were identified as activated CD69-positive T cells producing IFN-γ (see Extended Data Fig. 3 for gating strategy). Moreover, coexpression of IL-2 and TNF-α was analyzed to characterize cytokine expression profiles. Reactive CD4 and CD8 T cell levels after control stimulations were subtracted from those obtained after SARS-CoV-2-specific stimulation, and 0.03% of reactive T cells was set as the detection limit based on the distribution of T cell frequencies after control stimulations.

### Determination of SARS-CoV-2-specific antibodies and neutralization capacity

SARS-CoV-2-specific IgG antibodies towards the receptor binding domain of SARS-CoV-2 spike protein were quantified using an enzyme-linked immunosorbent assay (ELISA) according to the manufacturer’s instructions (SARS-CoV-2-QuantiVac, Euroimmun). Antibody binding units (BAU ml^–1^) <25.6 were scored negative, ≥25.6 and <35.2 were scored intermediate and ≥35.2 were scored positive. SARS-CoV-2-specific IgGs towards the N protein were quantified using ELISA according to the manufacturer´s instructions (Anti-SARS-CoV-2-NCP-ELISA, Euroimmun). Antibody levels are calculated as ratios defined as the extinction of the patient sample divided by the extinction of a calibrator serum. Ratios ≥1.1 were scored positive. A neutralization assay based on antibody-mediated inhibition of soluble ACE2 binding to the plate-bound S1 receptor binding domain was used at a single serum dilution according to the manufacturer’s instructions (SARS-CoV-2-NeutraLISA, Euroimmun). Surrogate neutralizing capacity was calculated as percentage of inhibition (IH) by 1 minus the ratio of the extinction of the respective sample and the extinction of the blank value. IH < 20% was scored negative, IH ≥ 20 to <35 intermediate and IH ≥ 35% positive.

### Statistical analysis

Kruskal–Wallis testing, followed by Dunn’s multiple comparisons test, was performed to compare unpaired nonparametric data between groups (lymphocyte subpopulations, T cell and antibody levels). Data with normal distribution were analyzed using ordinary one-way analysis of variance (cytokine expression profiles, age). Categorial analyses on gender and adverse events were performed using the *X*^2^ test. Correlations between levels of T cells, antibodies and plasmablasts were analyzed according to Spearman, and *P* < 0.05 was considered statistically significant. Analysis was carried out using GraphPad Prism 9.0 software using two-tailed tests. Cytokine profiles were plotted using the VennDiagram package (v.1.6.20)^21^ running under R (v.4.0.2).

### Reporting Summary

Further information on research design is available in the Nature Research Reporting Summary linked to this article.

## Online content

Any methods, additional references, Nature Research reporting summaries, source data, extended data, supplementary information, acknowledgements, peer review information; details of author contributions and competing interests; and statements of data and code availability are available at 10.1038/s41591-021-01464-w.

## Supplementary information

Supplementary Information Supplementary Tables 1 and 2 and Supplementary Fig. 1.

Reporting Summary

## Data Availability

Figures 1 and 2 and Extended Data Figs. 2 and 4–7 have associated raw data. The data that support the findings of this study are available from the corresponding author upon request, and data are available in a public repository (10.5281/zenodo.5080642). Because age and the individual assignment of the mRNA vaccine (BNT162b2 or mRNA-1273) may be subject to confidentiality, data in the repository refer to age groups, and mRNA vaccines are not specified individually.
