# Supplementary material for: Immunogenicity and reactogenicity of heterologous ChAdOx1 nCoV-19/mRNA vaccination
Source: Nat Med. 2021 Jul 26;27(9):1530–5. doi: 10.1038/s41591-021-01464-w (PMC8440177; doi:10.1038/s41591-021-01464-w)
Supplement: Supplementary file 1 — Supplementary Tables 1 and 2 and Supplementary Fig. 1. [file 41591_2021_1464_MOESM1_ESM.pdf]

nature portfolio

https://doi.org/10.1038/s41591-021-01464-w

Supplementary information

Immunogenicity and reactogenicity of heterologous ChAdOx1 nCoV-19/mRNA vaccination

In the format provided by the authors and unedited

Supplementary information

Immunogenicity and reactogenicity of heterologous ChAdOx1 nCoV-19/mRNA vaccination

Tina Schmidt[1], Verena Klemis[1,§], David Schub[1,§], Janine Mihm[2], Franziska Hielscher[1], Stefanie Marx[1], Amina Abu-Omar[1], Laura Ziegler[1], Candida Guckelmus[1], Rebecca Urschel[1], Sophie Schneitler[3], Sören L. Becker[3], Barbara C. Gärtner[3], Urban Sester[2], and Martina Sester[1;*]

[1]Department of Transplant and Infection Immunology, [2]Department of Internal Medicine IV, [3]Institute of Medical Microbiology and Hygiene, Saarland University, 66421 Homburg, Germany.

[§]these authors contributed equally

Supplementary Table 1: Test statistics for correlations of immune parameters in individuals on the three regimens.

	Vector/vector		Vector/mRNA		mRNA/mRNA	
	**R (CI)***	**P value***	**R (CI)***	**P value***	**R (CI)***	**P value***
CD4 vs CD8	0.343 (0.078-0.563)	0.010	0.336 (0.118-0.491)	0.002	0.421 (0.184-0.612)	0.0007
CD4 vs IgG	0.341 (0.754-0.562)	0.011	0.303 (0.103-0.479)	0.003	0.203 (-0.057-0.437)	0.114
CD4 vs Neutralizing ab	0.213 (-0.066-0.461)	0.122	0.301 (0.101-0.478)	0.003	0.297 (0.044-0.515)	0.019
CD4 vs Plasmablasts	-0.129 (-0.388-0.149)	0.347	-0.123 (-0.321-0.085)	0.233	-0.025 (-0.369-0.325)	0.889
CD8 vs IgG	0.060 (-0.216-0.328)	0.662	0.046 (-0.162-0.250)	0.659	0.406 (0.167-0.600)	0.001
CD8 vs Neutralizing ab	-0.092 (-0.358-0.188)	0.507	0.146 (-0.062-0.342)	0.155	0.365 (0.119-0.568)	0.004
CD8 vs Plasmablasts	-0.119 (-0.380-0.159)	0.388	0.074 (-0.135-0.276)	0.475	0.009 (-0.339-0.356)	0.958
IgG vs Neutralizing ab	0.891 (0.816-0.937)	<0.0001	0.565 (0.407-0.691)	<0.0001	0.569 (0.366-0.721)	<0.0001
IgG vs Plasmablasts	0.100 (-0.177-0.363)	0.466	-0.114 (-0.313-0.094)	0.269	0.023 (-0.327-0.368)	0.896
Neutralizing ab vs Plasmablasts	0.115 (-0.166-0.378)	0.408	-0.104 (-0.304-0.105)	0.315	0.145 (-0.213-0.469)	0.414

* two-tailed Spearman correlation; ab, antibody; R, correlation coefficient; CI, confidence interval.

Supplementary Table 2: Antibodies for flow-cytometric analyses

Antigen	conjugate	clone	isotype	reactivity	Catalogue number
CD3	PerCP	SK7	IgG1 k	mouse anti-human	345766
CD4	APC-H7	SK3	IgG1 k	mouse anti-human	641398
CD8	V500, PerCP	RPA-T8, SK1	IgG1 k	mouse anti-human	560774, 345774
CD19	FITC	HIB19	IgG1 k	mouse anti-human	555412
CD27	APC	L128	IgG1 k	mouse anti-human	337169
CD38	PE	HB7	IgG1 k	mouse anti-human	345806
CD69	PE-Cy7	L78	IgG1 k	mouse anti-human	335792
IFNγ	FITC	4S.B3	IgG1 k	mouse anti-human	554551
IgD	PE-Cy7	IA6-2	IgG2a k	mouse anti-human	561314
IL-2	PE	MQ1-17H12	IgG2a k	rat anti-human	559334
TNFα	V450	MAb11	IgG1 k	mouse anti-human	561311

All antibodies were purchased from BD.

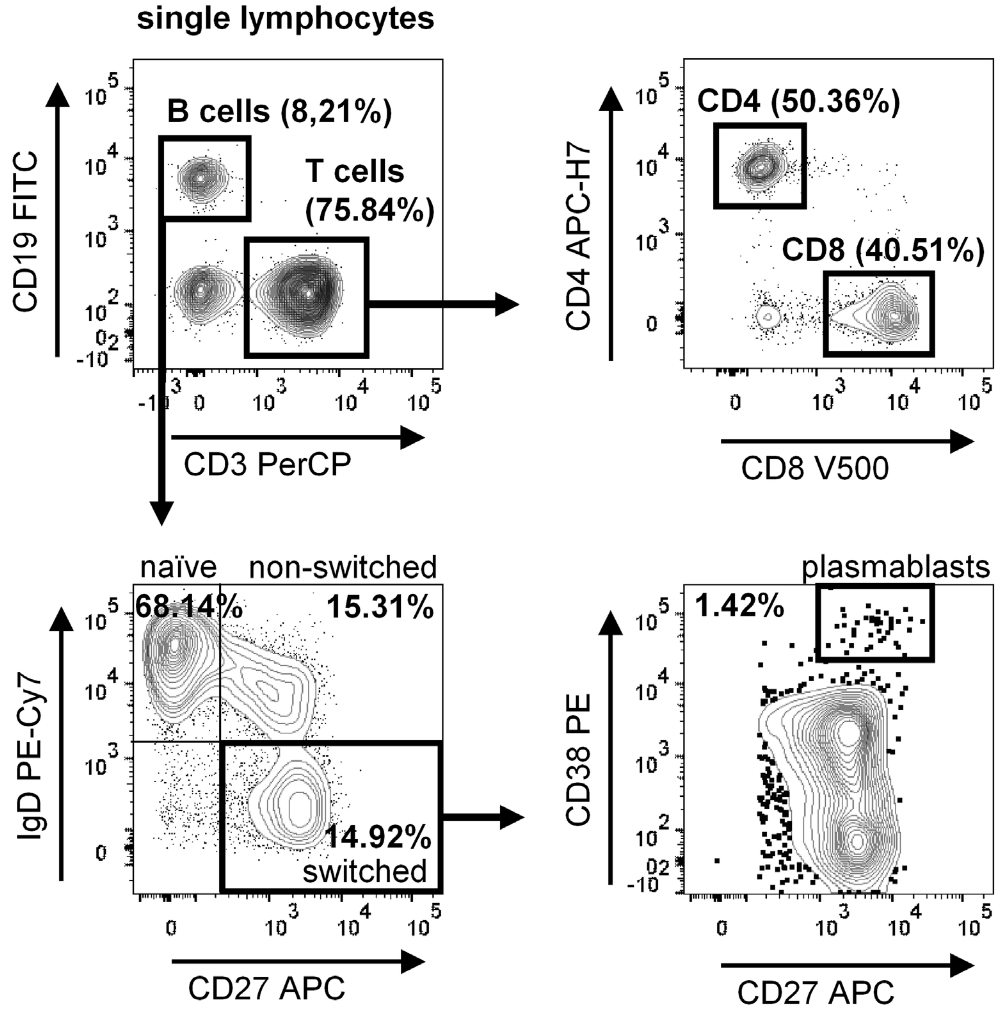

Supplementary Figure 1: Gating strategy for analysis of T-cells and plasmablasts. CD3 positive T-cells were identified among single lymphocytes and further subclassified in CD4 and CD8 T-cells. Among single lymphocytes, B cells were identified by CD19 expression and subclassified in naïve, non-switched memory and switched memory B cells according to expression of IgD and CD27. Plasmablasts were identified by high expression of CD38 among switched memory B cells.
